# Hyaluronan Metabolism is Associated with DNA Repair Genes in Breast and Colorectal Cancer. Screening of Potential Progression Markers Using qPCR

**DOI:** 10.3390/biomedicines8070183

**Published:** 2020-06-29

**Authors:** Ina Sevic, Fiorella Mercedes Spinelli, Daiana Lujan Vitale, Antonella Icardi, Lucia Romano, Alejandra Brandone, Paula Giannoni, Carolina Cristina, Marcela Fabiana Bolontrade, Laura Alaniz

**Affiliations:** 1 Laboratorio de Microambiente Tumoral; Centro de Investigaciones Básicas y Aplicadas (CIBA), CIT NOBA, Universidad Nacional del Noroeste de la Pcia. de Bs. As. Consejo Nacional de Investigaciones Científicas y Técnicas (UNNOBA-CONICET), Junín B6000, Argentina; isevic@comunidad.unnoba.edu.ar (I.S.); fspinelli@comunidad.unnoba.edu.ar (F.M.S.); dlvitale@comunidad.unnoba.edu.ar (D.L.V.); antoicardi@gmail.com (A.I.); 2 Laboratorio de Fisiopatología de la Hipófisis; Centro de Investigaciones Básicas y Aplicadas (CIBA), CIT NOBA, Universidad Nacional del Noroeste de la Pcia. de Bs. As. Consejo Nacional de Investigaciones Científicas y Técnicas (UNNOBA-CONICET), Junín B6000, Argentina; mluciaromano8@gmail.com (L.R.); carolina.cristina@nexo.unnoba.edu.ar (C.C.); 3 Hospital Interzonal General de Agudos Dr. Abraham F. Piñeyro, Junín B6000, Argentina; laulaniz13@gmail.com; 4 Clínica Centro, Junín B6000, Argentina; apgiannoni@comunidad.unnoba.edu.ar; 5 Instituto de Medicina Traslacional e Ingeniería Biomédica (IMTIB)-CONICET-Instituto Universitario del Hospital Italiano-Hospital Italiano Buenos Aires (HIBA), Buenos Aires C1199ACL, Argentina; marcela.bolontrade@hospitalitaliano.org.ar

**Keywords:** tumor microenvironment, hyaluronan, hyaluronan metabolism, breast cancer, colorectal cancer, qPCR

## Abstract

In this work, we compared mRNA levels of Hyaluronan (HA) metabolism members and BRCA genes, known to be involved in the tumoral process, between tumor and non-tumor adjacent tissue and its correlation with previously proposed biomarkers (ER, PR, HER2 and KI67) in order to assess their value as a progression biomarkers. We show alteration in HA metabolism in colorectal but not breast cancer. However, we found a decrease in Hyaluronidase 1 HYAL1 levels in the breast but not colorectal cancer. We also show lower HA levels in tumor compared with normal tissue that could indicate a possible influence of tumor on its surrounding “normal” tissue. In both breast and colorectal cancer, CD44 and BRCA2 showed a strong positive correlation. Besides, our results show first indicators that qPCR of the analyzed genes could be used as an easy and low cost procedure for the evaluation of molecular markers we propose here.

## 1. Introduction

Cancer is one of the leading causes of death worldwide. It is estimated that in 2012 there were 14.1 million new cases and 8.2 million deaths related to this disease. Approximately 70% of these cases occurred in developing countries. The most common types of cancer in men are lung, prostate, colorectal, stomach and liver, while breast, colon, lung, cervix and stomach cancer are the most common among women [1]. Mainly, breast, cervical and colorectal cancer are among the most common cancer types in Latin America [2]. The cancer burden continues to be a global problem. Many health systems in underdeveloped and developing countries are not prepared to manage this problem due to low-income and as a consequence large numbers of cancer patients do not have access to quality diagnosis and treatment [3].

At the moment of diagnosis, the correct designation of the cancer stage is the key for prognosis and appropriate treatment. The anatomical stages TNM (Anatomical extension of tumor—T, lymph nodes—N and metastasis—M) are considered the recommended way to specify different types of cancer [4,5]. However, it is evident that patients, even when they present the same stage of cancer, do not present the same disease development nor the prognosis or response to therapy. For this reason, it is favorable to include more factors that could help to define cancer subgroups. There are different proposals for defining subgroups. In the case of breast cancer, for example, the patients are divided into subgroups based on hormonal receptors [6,7,8]. In recent years, several tumor biomarkers have been identified that help to classify breast cancer into subgroups that have different prognoses and treatment: the estrogen receptor (ER), the progesterone receptor (PR) and the human epidermal growth factor receptor 2 (HER2) [9]. However, some biomarkers were proposed but never fully accepted for clinical use. Ki67 is a marker of cell proliferation, and it has been indicated that the Ki67 expression index independently predicts cancer progression. Also, since Ki67 is highly expressed in malignant cells, but detected in low expression in normal cells, it has been proposed as a biomarker for various types of tumors [10]. Here we investigate a couple more tentative markers that are highly associated with tumor progression and their association with already proposed ones.

During the past decade, tumors have been increasingly recognized as tissue whose complexity is similar or may even exceed that of healthy tissue. The heterogeneity and molecular complexity of cancer may explain, in part, the failure to effectively treat cancer patients [11]. The tumor microenvironment (TME) plays a significant role in the development of cancer and adds yet another factor that should be considered in the study of this pathology. TME is composed of cellular and non-cellular components that coexist in altered homeostasis. The main non-cellular component is the extracellular matrix (ECM), a complex network of macromolecules (proteins, glycoproteins, proteoglycans and glycosaminoglycans) with different biochemical properties and biological functions [12]. Among the components of the ECM that are altered in tumors is a glycosaminoglycan (GAG) Hyaluronic Acid or hyaluronan (HA). HA is a linear molecule composed of disaccharide units of N-acetylglucosamine and glucuronic acid. Several molecules are involved in its metabolism. HA is synthesized by specific synthetases (HAS): HAS1, HAS2 and HAS3. HA is degraded by enzymes called Hyaluronidases (HYAL) and also presents several isoforms. HA acts by inducing intracellular signals through several receptors: CD44, TLR4, LYVE1, RHAMM [13,14,15] that are also implicated in its metabolism. CD44 expression is usually associated with cancer stem cell characteristics and is considered a marker of these cells in most tumors [16,17]. Cancer stem cells (CSCs) are one of the cellular components of TME. These cells exhibit the capacity for self-renewal, pluripotency, high tumorigenic potential and resistance to therapy. Therefore, many cancer therapies, even though they eliminate most of the tumor cells, can ultimately fail because they do not eliminate the CSCs, which survive and regenerate new tumors [11,18,19]. CSCs are known to have constant interaction with their environment called niche. HA is closely associated with the stem cell niche. Several studies have reported a relationship between the level of HA and the invasiveness and aggressiveness of different tumors. It has been shown that the production of HA by stromal cells is stimulated by interactions with tumor cells, nonetheless, the synthesis also increases in the malignant tumor cells themselves. On the other hand, several members of the HA signaling pathway, like HA synthases (HAS1, HAS2, HAS3), HA receptors and hyaluronidases (mainly HYAL1) are critical determinants of growth and progression of the tumor [16,17,20]. Levels of the expression of CD44 and RHAMM have been associated with the progression of different types of cancer [14,21]. Taking this into account members of the HA signaling pathway could be considered as potential markers in a variety of carcinomas.

It has been shown that changes in the microenvironment can lead to changes in the expression of some genes. It is well known that BRCA 1 and 2 mutations are implicated in many types of cancer as a marker of susceptibility and are mainly associated with an increased risk of developing ovarian and breast cancer [22]. However, besides the BRCA mutation profile, there are indications that changes in the expression level of these genes could contribute to the tumor pathogenesis and could be associated with therapy response in sporadic cancer [23,24]. In our work, we evaluate the expression of BRCA 1 and 2 on the mRNA level to see it could be proposed as a predictor of tumor progression. The BRCA1 and BRCA2 genes have been widely studied in different types of tumors and it has been proven that changes in ECM can lead to the fixation of different mutations or the change in methylation pattern [25,26,27,28]. These events can lead to the change in the expression of the BRCA1 and BRCA2 proteins, which have an important role in the regulation of cellular processes and repair of damaged DNA. Also, the changes in these genes can influence the tumor microenvironment. All this leads to the creation of a feedback circle that increases the plasticity of tumor tissue and consequently to the progression of the disease [25,26]. On the other hand, it has been shown that RHAMM, one of the HA receptors, works in concert with BRCA1 to regulate the structure and polarization of normal breast cells as they grow. It is proposed that alteration in this relationship may, in turn, lead to the alteration of polarization in preneoplastic lesions [29].

It is considered that the number of cancer cases can be significantly reduced with the early detection and proper treatment of patients who develop cancer. In many cases there is a high probability of regression if it is diagnosed at the onset of the disease and treated in a patient-directed manner. For this reason, it is necessary to expand the knowledge of factors that affect the progression of cancer and the lack of success of therapy. Here we made the comparison among HA levels, HA metabolism pathway members and BRCA genes between tumor and non-tumor adjacent tissue and its correlation with previously proposed biomarkers in breast and colorectal cancer in order to assess their value as a potential progression biomarker. Considering that we proposed here a large number of potential markers, the main aim of this study was to select a set of the most promising biomarkers to be posteriorly evaluated in a bigger cohort of patients for the statistical confirmation of their utility.

## 2. Materials and Methods

### 2.1. Patients and Samples

Nine patients with colorectal and eight with breast cancer were selected for the analysis. The study included men and women over 18 years of age from the Surgery Department of Hospital Interzonal General de Agudos “Abraham Piñeyro” (HIGA) and Clinica Centro. The patients had previously signed an informed consent, approved (30.08.2018) by the ethics committee of the Hospital Austral, Province of Buenos Aires (17-006). Seven healthy donors (without malignancies, autoimmune or chronic diseases) were selected as control of plasma samples. This work was been carried out following The Code of Ethics of the World Medical Association. The investigations were carried out following the rules of the Declaration of Helsinki of 1975, revised in 2013.

Three types of samples were collected: tumor tissue (TT) discarded at the time of the surgery, non-tumor tissue adjacent to the tumor (NAT) and peripheral blood. Tissue specimens were collected in the operation room and were evaluated by a pathologist.

The focus of this study were patients with breast and colorectal cancer since these two cancers are considered to be the most frequent cancers in our region. Selected patients did not previously receive treatment for the current disease. Patients with an advanced stage of cancer or metastasis were excluded from this study. The colorectal cancer study involved nine patients (6 females and 3 males) with mean age 67.4 ± 10.3 years. For the breast cancer study, the patients were all female (8 patients) with mean age 61.3 ± 12.4 years. None of the colorectal cancer patients received radiotherapy or chemotherapy while two of the breast cancer patients received the therapy, 8 and 25 years ago, for another disease, and were restaged for this new tumor (Table 1). Histopathologic diagnosis for all the breast cancer patients was invasive carcinoma of no special type (NST), while for the colorectal patients was mostly adenocarcinoma of the colon. TNM stages were determined by a pathologist (Table 1).

### 2.2. Sample Processing

#### 2.2.1. Tumor and Non-Tumor Tissue

(a) Paraffin processing. In order to prepare tissue samples for immunohistochemistry, a part of tissue was fixed in 4% formaldehyde and included in paraffin. Briefly, tissue samples were dehydrated through a series of graded ethanol baths (70%, 96%, 100%) to displace the water, cleared with xylene and included in paraffin.

(b) RNA extraction. The tissue RNA was extracted using TRI reagent (Molecular Research Center, Inc., Cincinnati, OH). A DNAse treatment was performed in order to degrade contaminating DNA and afterward reverse transcription with Oligo (dT) primers (Genbiotech, CABA, Argentina) and M-MLV Reverse Transcriptase (M1701; Promega, Fitchburg, WI, USA) to obtain cDNA. Taking into account that RNA is easily degraded, in order to preserve it, before extraction, a preservation solution RNAhold (TransGen Biotech Co, Beijing, China) was used. RNA yield was determined by picodrop.

#### 2.2.2. Blood Samples

Whole blood pretreated with EDTA 4% was centrifuged for 15 min at 2000× *g* in order to separate plasma. Plasma samples were stored at −80 °C.

### 2.3. Quantification of RNA by Real Time PCR

To evaluate HA metabolism, the expression levels of hyaluronidases (HYAL1, HYAL2) and synthase (HAS2, HAS3) were analyzed. Also, CD44 RNA was analyzed in order to evaluate levels of one of the main HA receptors. On the other hand BRCA1 and BRCA2 RNAs were evaluated as the genes implicated in carcinogenesis.

Previously prepared cDNA was amplified by real-time PCR using Universal SYBR Green Supermix (1725271, Bio-Rad Laboratories, Hercules, CA, USA) and 200 nM of each specific primer (Invitrogen, Life Technologies, Carlsbad, CA, USA) (Table 2). PCR conditions were: 90 s at 94 °C and then 40 cycles of 30 s at 94 °C and 30 s at 60 °C. Relative levels of mRNAs were expressed as the “fold change” relative to the GAPDH gene. We used GAPDH as housekeeping gene considering that we never found much variability between our tumor samples.

### 2.4. Immunohistochemical Analysis

#### 2.4.1. Hematoxylin and Eosin Staining

To evaluate the state of the tumor and non-tumor tissue, Hematoxylin and Eosin staining was performed. Tissue sections were deparaffinized in xylene and were hydrated by passing through a decreasing concentration of ethanol baths (100%, 90%, 80%, 70%) and water. Afterwards, samples were stained in hematoxylin for 3 min and counterstained in 1% Eosin for 5 min. Stained sections were dehydrated and cleared in ethanol and afterward in xylene and mounted.

#### 2.4.2. HA Staining

HA staining was performed to determine the status of the extracellular matrix and to establish its level of expression in the tissue samples [30]. Briefly, tissue samples were deparaffinized in xylene and hydrated in decreasing concentrations of ethanol. Biotinylated HA-binding protein (bHABP) was used for this staining since there are no specific antibodies for HA. After an incubation O.N. at 4 °C, the samples were labeled with Streptavidin-FITC antibody to evaluate HA and DAPI to visualize the nuclei. To control the nonspecific signal in the tissues, a pretreatment control with hyaluronidase was made, in which no staining signal should be observed.

In order to obtain a semiquantification of positive area, 5–10 photographs per sample were obtained using a digital camera associated with a microscope (magnification ×200) and the average value of the marked area was calculated for each sample using ImageJ software. In the case of two samples which were very small, 5–6 photographs covered the entire area of the sample which made this number of photograps representative, while in all other cases the number of photographs was 10. DAPI was used for cell number normalization. The results were expressed as a “fold change” relative to non-tumoral tissue.

#### 2.4.3. ER, PR, HER2 and KI67 Staining

The expression levels of ER, PR, HER2 and KI67 in the case of breast cancer and the levels of KI67 in case of colorectal cancer were evaluated. Briefly, tissue samples were deparaffinized in xylene and hydrated in decreasing concentrations of ethanol. Afterwards, samples were treated with citrate (10 mM, pH = 6) for antigen retrieval. Endogenous peroxidase is blocked by incubation of tissue sections in 3% hydrogen. Samples with primary antibodies (249R-25 dilution 1:1, 323R-15 dilution 1:50, 237R-25 dilution 1:100 and 275R-15 dilution 1:200, Cell Marque, Santa Cruz, CA, USA) were incubated ON at 4C to improve the binding. The next day, samples were treated with HRP (Horseradish Peroxidase) conjugated secondary antibody (ab6720 dilution 1:200, Abcam, Cambridge, UK) and afterward revealed with DAB. HRP catalyzes the conversion of chromogenic substrates (DAB) into a colored product. Hematoxylin was used as a counterstain for better visualization and interpretation of tissue section. This analysis was done in Laboratory for High-level Technological services in CIBA, considering that hospitals in this region do not perform this analysis as a part of the diagnostics routine.

In the case of colorectal cancer, only KI67 was evaluated by counting positive cells and was expressed as positive against the total number of cells following proposed protocols for the evaluation of this marker in colorectal cancer.

In the case of breast cancer, all four markers were evaluated by a medical pathologist following standard diagnostic protocols established for diagnostics of breast cancer.

### 2.5. Plasma HA Analysis

HA levels in the plasma of patients and healthy donors were evaluated by ELISA-like assay established in our lab [31]. Briefly, the plate was coated with Hyaluronic Acid Binding Protein (HABP) after which the plate was incubated with plasma samples. Afterward Biotinylated Hyaluronic Acid Binding protein (b-HABP) was added and HA was revealed using streptavidin-HRP. The results were expressed in ng/mL and were obtained by extrapolation from the standard curve using linear regression analysis.

### 2.6. Statistical Analysis

GraphPad Prism was used for all statistical calculations. Student’s t-test was applied in all the cases where two variables were compared. Data are presented as the mean ± standard deviation. *P* values lower than 0.05 were considered significant. For the correlation analysis, the normality of data was assessed with Shapiro-Wilk test and Spearman´s correlation method was applied for the analysis. r-values higher than 0.8 (or lower than −0.8) were considered as a strong correlation and its statistical significance was evaluated by *p*-value. *P* values lower than 0.05 were considered significant.

## 3. Results

### 3.1. ER, PR, HER2 and KI67 Analysis

**Figure 1:**
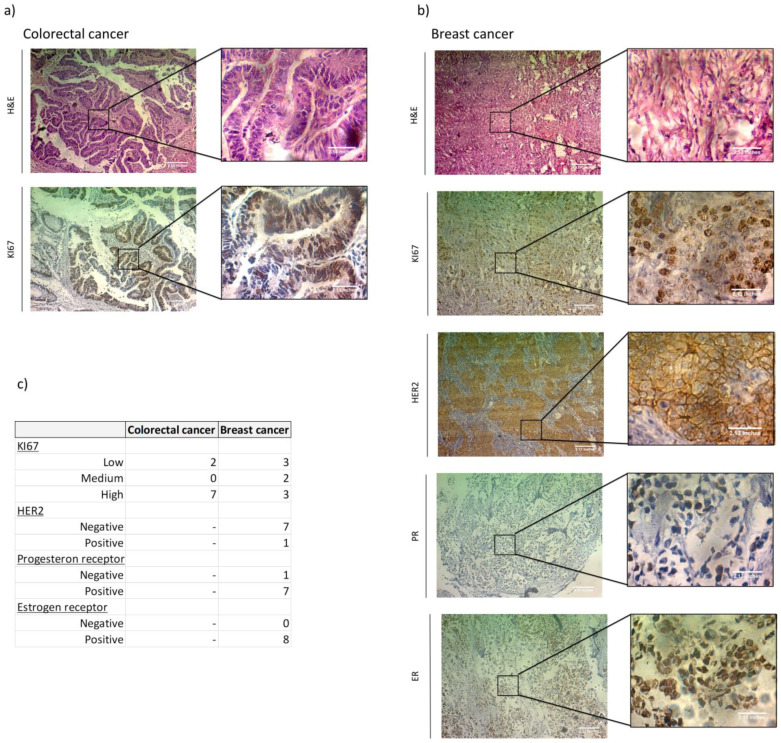
Immunohistochemical analysis of ER, PR, HER2 and KI67 in tumor tissue of colorectal and breast cancer patients. Representative images of positive ER, PR, HER2 and KI67 staining. Magnification 100× and 400×. (**a**) H&E and KI67 staining in colorectal cancer tissue. (**b**) H&E, KI67, HER2, PR and ER positive staining in breast cancer tissue. (**c**) Summary of the IHC staining results. The table shows the number of patients in each category. Dash shows a category that was not evaluated.

In order to establish the association between HA metabolism genes with previously proposed biomarkers, we first analyzed ER, PR, HER2 and KI67 in TT of the breast cancer patients and KI67 in TT of the colorectal cancer patients by immunohistochemistry (IHC). For the evaluation of KI67 nucloes positive cells, values less than 15% were considered low, 15-35% were intermediate and more than 35% were considered high. We found a high amount of KI67 positive cells in all (42%–63%) but two (7% and 9%) colorectal cancer patients, while in breast cancer we found three low (10%, 10% and 15%), two middle (30% and 35%) and three high (40%, 40% and 70%). All breast cancer patients were RE(+), all but one were RP(+) and only one was HER2(+). We had no triple negative patient subgroups (Figure 1, Table 3 and Table 4).

### 3.2. mRNA Analysis of Proposed Genes by qPCR

We evaluated mRNA of the genes involved in HA metabolism, specifically synthases: HAS2 and HAS3 and hyaluronidases: HYAL1 and HYAL2. We also analyzed CD44 mRNA as the main HA receptor that was described in ECM disorders in tumors. Lastly, we analyzed BRCA1 and BRCA2 mRNA because of their implications in cancer and DNA repair mechanisms. For this purpose, tissues obtained from surgical patients were processed, mRNA was extracted and analyzed as described in Section 2. All of the results were normalized to housekeeping gene GAPDH and shown as tumor tissue (TT) relative to non-tumor tissue adjacent to tumor (NAT).

**Figure 2:**
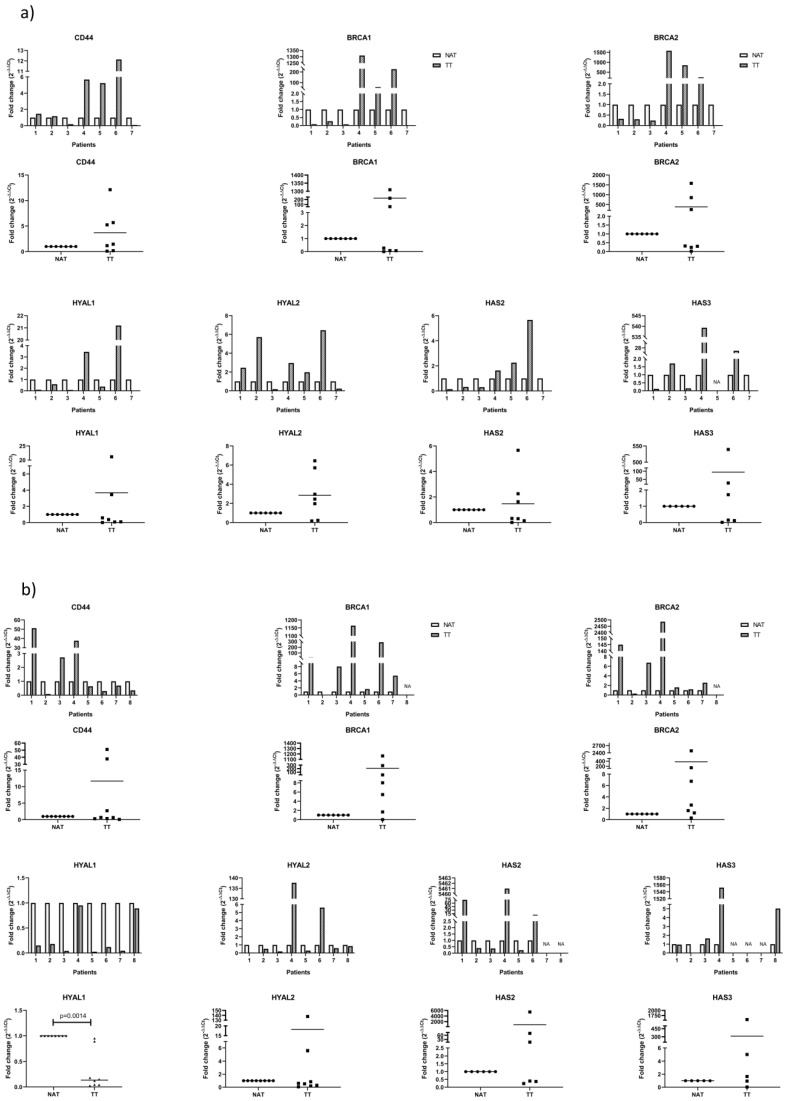
Real Time PCR analysis of the genes involved in HA metabolism and DNA repair. Results were normalized to housekeeping gene GAPDH and shown as tumor tissue (TT ▪) relative to non-tumor tissue adjacent to tumor (NAT ●). A P-value lower than 0.5 was considered statistically significant. NA indicates values that could not be obtained. (**a**) mRNA analysis of the tumor (TT) and adjacent non-tumor tissue (NAT) of the colorectal cancer patients. (**b**) mRNA analysis of the tumor (TT) and adjacent non-tumor tissue (NAT) of breast cancer patients.

In colorectal cancer samples, we found that none of the analyzed genes were consistently higher or lower in TT compared to NAT. However, we noticed that there is a group of patients (patients 4, 5 and 6) that have consistently different values for all evaluated genes, mostly higher, compared to the rest of the group (patients 1, 2, 3 and 7). HYAL1 is lower in all but two patients (patients 4 and 6) while HYAL2 is higher in all but two patients (patients 3 and 7) (Figure 2a, Table 3).

In breast cancer samples, the only gene that has consistently lower mRNA expression in TT is HYAL1 (*p* = 0.0014). HYAL2 decreased in all but two patients (patients 4 and 6). These two patients are the only in the group that went through radiotherapy in the past. BRCA 1 and 2 increase in TT in all but one patient (patient 2) which is the only patient in our study with RP- and HER2+ status. The rest of the genes showed high variability among patients. The only patient that showed consistently very high values for all genes was patient 4 (Figure 2b, Table 4). In the case of breast cancer, the most promising gene seams to be HYAL1 while BRCA1 and 2 could be considered for further testing in different groups in order to test if it depends on the HER2 or RP status of the patient.

### 3.3. Analysis of HA Levels in Plasma and Tissue

After analyzing mRNA levels of the genes involved in HA metabolism, we analyzed HA levels in order to test its association with HA accumulation in tumoral and its surrounding tissue. As a next step, we evaluated plasma HA levels to see does the previously observed in tissue reflects on plasma levels. We analyzed HA levels in TT and NAT. Considering that TT and NAT have different tissue architecture and as a consequence, different cell numbers and size, we normalized all measured values with DAPI as a marker of cellular nucleus, and therefore of cell number. All the results are expressed as TT relative to NAT. On the other hand, we evaluated HA levels in the plasma of cancer patients and healthy donors as a control group in order to obtain a value that reflects HA accumulation in tissues. In the case of colorectal cancer, there were seven healthy donors (5 female and 2 male), while in the case of breast cancer, all five healty donors were female.

**Figure 3:**
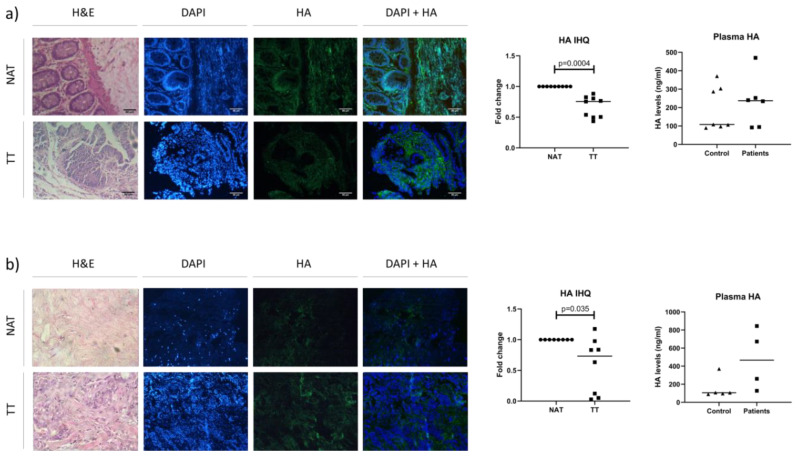
Tissue and plasma HA levels in patients with colorectal and breast cancer. (**a**) Colorectal cancer HA analysis. Representative image of immunohistochemical staining of HA (left) with the graph (middle) and plasma HA analysis of patients and healthy donors by ELISA (right). (**b**) Breast cancer HA analysis. Representative image of immunohistochemical staining of HA (left) with the graph (middle) and plasma HA analysis of patients (▪) and healthy donors (▲) by ELISA (right). H&E-hematoxylin and eosin; HA-hyaluronic acid; TT (▪)-tumor tissue; NAT (●)-adjacent non-tumor tissue. Results of immunohistochemistry are expressed as TT relative to NAT. p-values lower than 0.05 were considered statistically significant; Scale bar = 80 µm.

In both types of cancer, HA levels were significantly lower in TT comparing to NAT (colorectal cancer: *p* = 0.0004; breast cancer: *p* = 0.035). Only one patient presented higher HA levels in TT than NAT and it was the only T1 stage (breast cancer) patient in this study. Interestingly, serum HA levels were higher in patients compared to the control group, in both types of cancer, although not statistically significant (Figure 3).

### 3.4. Correlation Analysis of Proposed Markers

All of our analyses were evaluating the increase or decrease of an individual marker in tumor tissue comparing to healthy tissue. However, this is not necessarily always the case. Some reports show that some values can increase or decrease together forming groups. We performed a correlation analysis in order to test this in our group of patients and determine possible markers with prognostic values associated with HA metabolism and DNA repair mechanisms. For colorectal cancer, all mRNA, tissue HA and KI67 results were included in the correlation analysis, while for breast cancer, ER and PR were also included. HER2 was excluded from this analysis considering we only had one positive patient. The results were shown as a heat map with r values. All r values above 0.8 (or under −0.8) were considered as a strong correlation and its statistical significance was defined with the *p* value.

**Figure 4:**
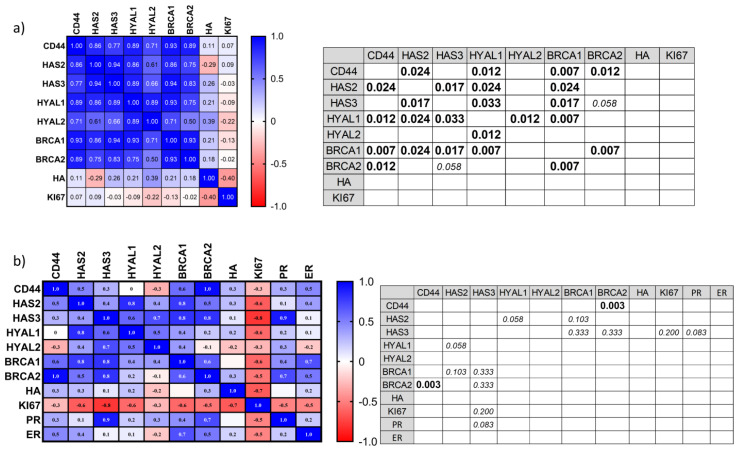
Correlation analysis of colorectal and breast cancer markers. (**a**) The results of correlation analysis for colorectal cancer are shown as a heat map with r values. All r values above 0.8 (or under −0.8) were considered as a strong correlation (left) and its statistical significance was defined with the *p* value shown in the table (right). (**b**) The results of correlation analysis for breast cancer are shown as a heat map with r values. All r values above 0.8 (or under −0.8) were considered as a strong correlation (left) and its statistical significance was defined with the *p* value shown in the table (right). *P*-values lower than 0.05 were considered statistically significant and are represented in bold text; *P*-values higher than 0.05 (with strong correlation result) are represented in italic text.

The results for colorectal cancer show positive correlation among mRNA of all analyzed genes, although only the following were statistically significant: CD44 correlated positively with HAS2 (*p* = 0.024), HYAL1 (*p* = 0.012), BRCA1 (*p* = 0.007) and BRCA2 (*p* = 0.012); HAS2 correlated positively with HAS3 (*p* = 0.017), HYAL1 (*p* = 0.024) and BRCA1 (*p* = 0.024); HAS3 correlated positively with HYAL1 (0.033) and BRCA1 (0.017); HYAL1 correlated positively with HYAL2 (*p* = 0.012) and BRCA1 (*p* = 0.007); BRCA1 correlated positively with BRCA2 (*p* = 0.007). HA and KI67 did not correlate significantly with any of the compared values (Figure 4a). This could indicate that in the case of colorectal cancer, there is a change in HA metabolism rather than just in one gene of the HA metabolism. Also, BRCA 1 and 2, as well as CD44, seam to be correlating with the changes in HA metabolism.

In the case of breast cancer, CD44 and BRCA2 showed a strong positive correlation (*p* = 0.003). When compared, mRNA values for breast cancer did not show the same correlation tendency as for colorectal cancer. HA, as in the previous case, did not correlate with any of the values. We also observed that PR showed a strong positive correlation with HAS3 and ER with CD44, while, KI67 correlated negatively with all other markers we tested. These last correlations were not statistically significant. However, considering that the *p*-value is very sensitive to sample number while r-value is not, we can suggest that high correlations we observed are important and should be further investigated in a larger cohort study (Figure 4b).

## 4. Discussion

Cancer is one of the most complex diseases we are facing today. It shows great cellular and molecular complexity, which makes the treatment and prediction of the disease progression very difficult. Cancer staging is usually the first and one of the most important steps for defining different tumors and for grouping patients according to their prognosis. For some tumors, the size of the tumor can be a key factor (like for breast cancer), while for colorectal patients, it has little impact on prognosis and the depth and extent of invasion are the primary prognostic features. TNM staging is widely accepted category criteria for defining the anatomic extent of tumor that defines prognosis and is a critical element in determining appropriate treatment [4]. In some parts of the world where tumor biomarker testing is not routinely done, TNM is used as the only tool to define the tumor grade and to group patients according to tumor stage.

However, it is increasingly evident that the patients, even if they have the same stage, do not present the same disease development which highlights the necessity for defining subgroups.

One of the most accepted subgroup divisions is in the case of breast cancer. The division here is made according to the ER, PR and HER2 gene expression and is included in the clinical and pathological prognostic grouping. These subgroups show significant differences in the disease treatment and response to therapy [8]. Onitilo et al. showed that triple negative breast cancer has a poor prognosis and the worst survival rate compared to other subgroups. Also, they show that triple negative and HER2+ER- subgroups have poorer clinical and pathological prognosis [32]. Kast et al. confirmed the elevated risk for metastasis of HER2+ and triple negative breast cancer [33]. Howlader et al. showed that triple-negative, ER+HER2+ and ER-HER2+ breast cancers carry a higher risk of mortality compared with ER+HER2- tumors [6]. However, Parise et al. show that T1a stage node-negative tumors have the same risk of mortality regardless of subtype and that being ER- and PR- shows stronger influence in patient survival than being HER2+ for tumors of all sizes [7]. According to these reports, most of our patients are in a lower mortality risk group (ER+HER2-) except for one patient (T2N2a ER+HER2+). However, patients described by different authors, even though they belong to the same subgroup, do not show the same disease progression and the necessity for molecular subgrouping is increasingly evident. This is especially important in order to obtain a predictive or prognostic indicator of relapse after surgery or treatment.

Proliferation is a hallmark of cancer, and Ki67 evaluation by immunohistochemistry is currently a valuable assay for measuring and monitoring tumor proliferation in tumoral tissue samples [34]. KI67 has been proposed for the prognosis and prediction of treatment response in both breast and colorectal cancer [35,36]. Since the variability of data obtained from the histological methods, which are dependent on the laboratory, reagents and operator that performs it, KI67 is still not considered a standard procedure for evaluating cancer subgroups. However, many studies have recognized its value as a predictive factor. Proliferation assessment is reported to be very important in ER+HER2– breast cancer for guiding the choice of treatment. It was shown that patients with KI67 >20% had the poorest disease-free interval and disease-specific survival [37,38]. Some groups report its high correlation with tumor stages while other report lack of correlation. There is increasing evidence that KI67 is a valuable predictive and prognostic marker. Its utility was demonstrated in measuring the response to certain adjuvant treatments and it was associated with a higher probability of relapse. Similarly, in colorectal cancer patients, it has been demonstrated that high KI67 expression correlates significantly with poor overall survival and disease-free survival cancer [35,36]. Even though different authors do not agree about the exact cut off value, they suggest that a Ki67 level above 15–20% defines a high-risk group for the disease prognosis [39]. Taking this into account, in our breast cancer patients as well as colorectal cancer patients we identified two groups: high and low risk. Our results show no correlation of KI67 groups with any of the measured values. On the other hand, if we evaluate individual KI67 values instead of dividing it into groups, we found in the breast but not colorectal cancer the tendency for the negative correlation with all of the measured values. According to our results, KI67 has more clinical value when observed as an individual value instead of division in a low and high risk group. However, a negative correlation with HAS3 in breast cancer could be an interesting marker to continue the study. This result is not statistically significant, which might be due to the small sample number and should be further investigated in a larger cohort study. Importantly, HAS3 enzyme produces HA of low (LMW) or middle (MMW) and is associated with metastasic behavior in some tumor cell lines [40,41]. Besides, it has been reported that this enzyme is the most active and predominant in pathological conditions [42].

It has been shown that the production of HA by stromal cells is stimulated by interactions with tumor cells, but that the synthesis also increases in the malignant tumor cells themselves. Hyaluronan synthesis and degradation are strictly regulated in physiological conditions, but in the tumoral tissues they are dysregulated, originating HA of different molecular sizes, which in turn have different functions [42]. Even more, HA interaction with specific binding proteins and receptors modulating its function in tumoral context [30]. It is well known that members of the family of molecules of the HA signaling pathway, like HA synthases (HAS1, HAS2, HAS3), HA receptors and hyaluronidases (mainly HYAL1) are critical determinants of tumor growth and progression [16,17,20]. CD44 and RHAMM expression levels have been linked to the progression of several types of cancer and are known to mediate HA cellular singnaling [14,15,21]. When comparing TT and NAT, we noticed a high variability of HAS2, HAS3 and HYAL2 among the patients for both tumor types while HAS1 and HYAL3 could not be detected. There are several reports indicating the role of HAS1 in the synthesis of HA, especially in animal models [43]. Even so, we could not detect HAS1 mRNA in any of the tissues in our experimental conditions. Nevertheless, the level of its expression in tumor is controversial. The lack or HAS1 expression could be compensated by the others isoenzyme HAS2 or HAS3, which even have a greater affinity for the substrate, HA. Rilla et al. showed that HAS1 is almost inactive in cells with low UDP-sugar levels and HAS2 activity increases with UDP-sugars while HAS3 produced HA at high speed even with minimum substrate content [44]. However, in the case of HYAL1, we found that mRNA levels significantly decrease in TT in breast cancer respect to NAT, while in colorectal cancer there is a tendency for HYAL1 to decrease in NAT respect to TT although not statistically significant. However, it is worth noting that there is differential behavior observed between the tumors. The behavior we observed for HAS is consistent with results previously reported by Tammi et al. where they report that HAS1, HAS2 and HAS3 mRNA levels show high variability among patients as they detect increase as well as a decrease in its levels and proposes that HA synthesis could be stimulated at the post-transcriptional level [45]. Some reports show that the expression of HYAL1 and HYAL2 depends highly on the cancer type. The function of HYAL1 was proposed to be as tumor suppressor as well as promoter. Its higher expression was reported in prostate, colon and breast cancer and lower expression in lung cancer. This could be associated with the breakdown of HA, generated by LMW HA promoting angiogenesis and invasion [45]. Nykopp et al. reported decreased HYAL1 mRNA in ovarian and endometrial carcinoma and associated it with stromal HA accumulation [46]. The same group also shows that even though they observed reduced HYAL1 expression, HAS levels were not consistently elevated [47]. On the other hand, Tan et al. report elevated levels of HYAL1, HYAL2 and HA in breast cancer and show that breast cancer cells with higher hyaluronidase expression exhibit significantly higher invasion ability in matrigel [48].

Many reports show that an increase in HA levels in tumor ECM correlates with tumor progression of different type of cancer, as carcinoma, sarcoma, ect. [45,49]. The loss or decrease of HYAL1 was proposed as one of the reasons. The loss of HYAL1 can happen on DNA, RNA or protein levels. Considering that we found the decrease in mRNA levels indicates that it could be at the genomic DNA level. Some authors consider that the loss could happen at the translation level or at the protein inactivation level [50]. Also, some reports show that HA content does not correlate with the mRNA levels of the HA synthases, HAS1, HAS2 or HAS3 [51]. This could mean that the loss of HYAL1 as well as an increase in HA could be a great candidate for tumor markers. However, our results show that although we saw a decrease in HYAL1 in tumor tissue, we also saw a decrease in HA levels. Thus, an increase or decrease of HYAL1 might not be associated with a change in total HA accumulation. Rather, it would seem to be associated with functional changes in the HA molecule and, as a consequence changes in HA MW. On the other hand, the level of HA does not necessarily depend on an increase or decrease in HYAL1 and its metabolism is more complexly regulated by posttranscriptional events. However, considering that we found different patterns of expression of this molecule between tumors and also between TT and NAT tissue, makes it an important target for studying and defines it as a possible independent marker of progression.

In addition to the complexity of the HAS or HYAL regulation at transcriptional or postranscriptional level, the activity rate of this enzymes is associated with different metabolic routes, beyond their expression level. For example, UDP-sugar substrates derived from high glycolytic activity impact hyaluronan synthesis in tumor or surrounding tissues [52,53]. Higher HA levels in surrounding normal tissue compared to tumor tissue are consistent with previously observed by Josefsson et al., who reported the increase in HA staining score in morphologically normal prostate tissue that surrounds the tumor. They also report that increased HA staining score in nonmalignant tissue was prognostic in low stage prostate tumor and propose that in negative biopsies, HA levels could be used to indicate the risk that aggressive tumor could be present elsewhere in the prostate [54]. Taking into account that our results show higher HA levels in healthy tissue surrounding the tumor could mean that it could be used as a molecular prognostic marker that shows the presence of a tumor close by. Our results of plasma HA in the breast, as well as colorectal patients comparing to healthy donors, show the tendency to increase, although not statistically significant. These results are in accordance with previous reports [55] and could indicate, in some cases, the presence of a tumor that is in progression.

It has been previously reported in different tumors that the expression of CD44 increases in tumor tissue and is associated with tumor progression [56]. Our results show inconsistencies in CD44 expression in both, breast and colorectal carcinoma patients. Even though the levels of CD44 and HA can be increased in several types of malignant tumors, in some cancers, its levels are not a consistent indicator of unfavorable prognosis [45].

Even though we did not observe consistently lower or higher levels of HAS, HYAL2 or CD44 correlativity tests for colorectal cancer show a strong positive correlation between HAS, HYAL and CD44; many of them with strong statistical significance. This was not the case for breast cancer patients. This result can indicate that in colorectal, but not breast cancer, HA metabolism in the tumor tissue tends to up regulate or down regulate in its entirety, showing the alteration in entire metabolism rather than just one molecule. However, we have previously observed that the effect of HMW HA as an inductor of the angiogenic behavior of macrophages in breast cancer is in part consequence of the presence of TSG-6 [30]. This observation could indicate that the function of HA, more than its metabolism, could be altered in the case of breast cancer. Thus, the expression of different associated molecules, like TSG-6, could explain the results we observed in breast cancer models and patients. Indicating that HA is a molecule that favors tumor progression by its differential function more than its accumulation or metabolism.

Many reports show that normal tissue adjacent to the tumor does not have all the signatures of the normal tissue and possibly suffers the influence of the tumor cells from its vicinity [54]. Here we saw this behavior in the case of tissue HA levels but also in the case of some of colorectal and breast cancer patients for the expression of BRCA1 and BRCA2. BRCA1 and BRCA2 are tumor suppressor genes that encode for proteins that maintain genomic stability through DNA repair. Cells that have BRCA mutations cannot repair the DNA damage that appears because of continuous cell cycles, which typically results in cell death [28]. The influence of mutations in these genes on cancer risk has been described for many cancers including breast, prostate, colorectal and stomach cancer [27,28,57]. However, the expression levels of these genes also affect the DNA repair systems. For example, Tsibulak et al., observed that low BRCA1/2 mRNA expression confers platinum-hypersensitivity in ovarium cancer [23]. Also, BRCA1 and 2 mRNA and protein expression levels are proposed as prognostic biomarkers [58]. In concordance with these observations, our results show that if there is an increase or decrease in BRCA1 the same is observed for BRCA2, which could indicate that there is an alteration in the DNA repair system of that tissue. However, we observed this in the tumor as well as healthy adjacent tissue. Atkinson et al. show that in patients with triple negative breast cancer stem cells are enriched in normal adjacent tissue while in ER+ and HER2+ this was observed only in some patients. They obtained similar results for the alteration in the DNA repair system in normal adjacent tissue [59]. Wang et al. observed p53 alteration in benign-appearing breast glands in patients with high-grade breast tumor (55%) while in low-grade they still observe this behavior in some patients (6.25%) [60]. This finding could explain, at least in part, the BRCA1 and BRCA2 discrepancies in healthy compared to tumor tissue we observe in some of the patients. Our results show a positive correlation between BRCA1 and CD44, HAS2, HAS3 and HYAL1 in colorectal but not breast cancer. On the other hand, BRCA2 correlated with CD44 in both types of cancer. As far as we know, this is the first report on the correlation between BRCA and HA metabolism genes. Further investigation would be necessary to clarify this connection as well as its biological implication.

In summary, we show an alteration in mRNA of genes associated with HA metabolism in the colon in correlation analysis, while in breast cancer we observed a decrease in HYAL1 levels. We also show lower HA levels in tumor comparing with normal tissue that could indicate a possible influence of tumor on its surrounding normal tissue. Our results show the selection of the most promising molecules that should be further investigated in a larger cohort study to confirm their utility as a molecular marker for the tumor progression using these methods.

Also, as our objective is to provide a method of easy access for oncologic patients in low- income countries we used qPCR in our study as a highly available method that will allow the adequate analysis of prognostic factors and, therefore, the proper treatment. We aimed to add to the solving of the problem of a large number of cancer patients who do not have access to quality diagnostics and treatment.

Finally, we consider that this study sets an adequate background for further investigations of these genes that could provide valuable prognostic information and further improve the clinical prediction of the progression of breast and colorectal cancer patients.

## Figures and Tables

**Table 1:** Patient data.

Patient Characteristics	Breast Cancer	Colorectal Cancer
Number of patients	8	9
Average age ± SD, years	61.3 ± 12.4	67.4 ± 10.3
Gender, Male/Female	0/8	6/3
Prior treatment history:		
Chemotherapy	1	0
Radiotherapy	2	0
Tumor size		
T1	1	0
T2	6	0
T3	1	4
T4	0	2
Unknown	0	3
Lymph node status		
N0	2	3
N1	1	3
N2	2	0
N3	1	0
Unknown	2	3
Metastasis	0	0

**Table 2:** List of primers used in this study.

Primer	Primer Direction	Sequence
GAPDH	Forward	5′-GGGGCTGCCCAGAACATCAT-3′
	Reverse	5′-GCCTGCTTCACCACCTTCTTG-3′
HAS2	Forward	5′-TACACAGCCTTCAGAGCACTG-3′
	Reverse	5′-ATGAGGCTGGGTCAAGCATAG-3′
HAS3	Forward	5′-TGCACCATCGAGATGCTTCG-3′
	Reverse	5′-CCATGAGTCGTACTTGTTGAGG-3′
HYAL1	Forward	5′-GGCTATGAGGAAACTGAGTCAC-3′
	Reverse	5′-TAGGAGTGCAAGGGCTGTAC-3′
HYAL2	Forward	5′-ATCTCTACCATTGGCGAGAGTG-3′
	Reverse	5′-ATCTTTGAGGTACTGGCAGGTC-3′
CD44	Forward	5′-GTGATGGCACCCGCTATG-3′
	Reverse	5′-ACTGTCTTCGTCTGGGATGG-3′
BRCA1	Forward	5′-GGCTATCCTCTCAGAGTGACATTT-3′
	Reverse	5′-GCTTTATCAGGTTATGTTGCATGG-3′
BRCA2	Forward	5′-CCAAGTGGTCCACCCCAAC-3′
	Reverse	5′-CACAATTAGGAGAAGACATCAGAAGC-3′

**Table 3:** Summary of the results for the colorectal tumor patients.

Patient	Sex	Age at Sample Acquisition (yr)	Histopathologic Diagnosis	TNM Stage	Chemotherapy	Radiotherapy	KI67	HA	CD44	HAS2	HAS3	HYAL1	HYAL2	BRCA1	BRCA2
1	F	58	Adenocarcinoma of caecum	pT4pN1b	NO	NO	62%	↓	↑	↓	↓	↓↓	↑	↓↓	↓
2	M	58	Villous adenoma with high grade dysplasia	NA	NO	NO	43%	↓	↑	↓	↑	↓	↑	↓	↓
3	M	67	Adenocarcinoma of colon	NA	NO	NO	63%	↓	↓	↓	↓	↓↓	↓	↓↓	↓
4	M	64	Adenocarcinoma of colon	pT3pN1	NO	NO	42%	↓	↑	↑	↑↑↑	↑	↑	↑↑↑	↑↑↑
5	M	65	Adenocarcinoma of colon	pT4pN0	NO	NO	44%	↓	↑	↑	NA	↓	↑	↑↑	↑↑↑
6	F	74	Adenocarcinoma of colon	pT3pN0	NO	NO	44%	↓	↑↑	↑	↑↑	↑↑	↑	↑↑↑	↑↑↑
7	M	57	Adenocarcinoma of colon	NA	NO	NO	7%	↓	↓↓	↓↓↓	↓↓	↓↓↓	↓	↓↓↓	↓↓
8	F	76	Adenocarcinoma of colon	pT3pN1	NO	NO	49%	↓	NA	NA	NA	NA	NA	NA	NA
9	M	88	Adenocarcinoma of colon	pT3pN0	NO	NO	9%	↓	NA	NA	NA	NA	NA	NA	NA
	6M/3F	67.4 ± 10.3													

Note: Arrows indicate an increase ↑ or decrease ↓ of the value measured in tumor tissue compared with adjacent non-tumor tissue. Values lower than 10 fold are indicated with one arrow, between 10 and 100 fold with two arrows and values higher than 100 fold with three arrows. NA indicates values that could not be obtained.

**Table 4:** Summary of the results for breast cancer patients.

Patient	Sex	Age at sample Acquisition (yr)	Histopathologic Diagnosis	TNM Stage	Chemotherapy	Radiotherapy	RE	RP	HER2	KI67	HA	CD44	HAS2	HAS3	HYAL1	HYAL2	BRCA1	BRCA2
1	F	53	Invasive carcinoma of no special type (NST)	pT1 pNo	NO	NO	**98% intense**	**10%**	**NEG**	**15%**	↑	↑↑	↑↑	↓	↓	↓↓	↑↑	↑↑↑
2	F	63	Invasive carcinoma of no special type (NST)	T2pN2a	NO	NO	**30% intense**	**NEG**	**POS 3+**	**40%**	↓	↓↓	↓	↓↓	↓	↓	↓↓	↓
3	F	61	Invasive carcinoma of no special type (NST)	pT2pNo	NO	NO	**95% intense**	**70%**	**NEG**	**40%**	↓↓	↑	↓	↑	↓↓	↓	↑	↑
4	F	75	Invasive carcinoma of no special type (NST)	pT2pNx	NO	*	**90% moderate**	**90%**	**NEG**	**10%**	↓	↑↑	↑↑↑	↑↑↑	↓	↑↑↑	↑↑↑	↑↑↑
5	F	35	Invasive carcinoma of no special type (NST)	pT3pN3a	NO	NO	**70% intense**	**30%**	**NEG**	**30%**	↓	↓	↓	NA	↓↓	↓	↑	↑
6	F	69	Invasive carcinoma of no special type (NST)	T2Nx	*	*	**95% intense**	**10% intense**	**NEG**	**35%**	↓↓	↓	↑↑	NA	↓	↑	↑↑↑	↑
7	F	69	Invasive carcinoma of no special type (NST)	pT2N2a	NO	NO	**35% moderate**	**30% moderate**	**NEG**	**70%**	↓	↓	NA	NA	↓↓	↓	↑	↑
8	F	65	Invasive carcinoma of no special type (NST)	T2pN1	NO	NO	**95% intense**	**95% intense**	**NEG**	**10%**	↓	↓	NA	↑	↓	↓	NA	NA
		61.3 ±12.4																

Note: Arrows indicate an increase ↑ or decrease ↓ of the value measured in tumor tissue compared with adjacent non-tumor tissue. Values lower than 10 fold are indicated with one arrow, between 10 and 100 fold with two arrows and values higher than 100 fold with three arrows. NA indicates values that could not be obtained. * indicates patients who underwent therapy in the past for another disease (patient 4, 25 years ago and patient 6, 8 years ago).
